# Is Boundary Annotation Necessary? Evaluating Boundary-Free Approaches to Improve Clinical Named Entity Annotation Efficiency: Case Study

**DOI:** 10.2196/59680

**Published:** 2024-07-02

**Authors:** Gabriel Herman Bernardim Andrade, Shuntaro Yada, Eiji Aramaki

**Affiliations:** 1 Graduate School of Science and Technology Nara Institute of Science and Technology Ikoma Japan

**Keywords:** natural language processing, named entity recognition, information extraction, text annotation, entity boundaries, lenient annotation, case reports, annotation, case study, medical case report, efficiency, model, model performance, dataset, Japan, Japanese, entity, clinical domain, clinical

## Abstract

**Background:**

Named entity recognition (NER) is a fundamental task in natural language processing. However, it is typically preceded by named entity annotation, which poses several challenges, especially in the clinical domain. For instance, determining entity boundaries is one of the most common sources of disagreements between annotators due to questions such as whether modifiers or peripheral words should be annotated. If unresolved, these can induce inconsistency in the produced corpora, yet, on the other hand, strict guidelines or adjudication sessions can further prolong an already slow and convoluted process.

**Objective:**

The aim of this study is to address these challenges by evaluating 2 novel annotation methodologies, *lenient span* and *point annotation*, aiming to mitigate the difficulty of precisely determining entity boundaries.

**Methods:**

We evaluate their effects through an annotation case study on a Japanese medical case report data set. We compare annotation time, annotator agreement, and the quality of the produced labeling and assess the impact on the performance of an NER system trained on the annotated corpus.

**Results:**

We saw significant improvements in the labeling process efficiency, with up to a 25% reduction in overall annotation time and even a 10% improvement in annotator agreement compared to the traditional boundary-strict approach. However, even the best-achieved NER model presented some drop in performance compared to the traditional annotation methodology.

**Conclusions:**

Our findings demonstrate a balance between annotation speed and model performance. Although disregarding boundary information affects model performance to some extent, this is counterbalanced by significant reductions in the annotator’s workload and notable improvements in the speed of the annotation process. These benefits may prove valuable in various applications, offering an attractive compromise for developers and researchers.

## Introduction

### Overview

The electronic health record (EHR) can be an important source of data for health-related research as it contains information on a patient’s condition and complaints, performed procedures and administered drugs, the outcome of the treatment, and more [1].

Clinical narratives are a fundamental part of EHRs. Due to their free and unstructured format, natural language processing (NLP) methods are essential for extracting the information from such documents in a way that is comprehensible and useful for computer systems. Although machine learning–based NLP systems can achieve high performance, these often require large amounts of in-domain annotated data for proper training [2]. Recent few-shot approaches empowered by large language models (LLMs) have also been shown to be performant. Yet, these can also benefit from fine-tuning with in-domain examples, yielding notable improvements [3].

Named entity (NE) annotation, as an inherently manual process, allied to the sheer volume of data that must be meticulously labeled to produce an accurate model, makes it an exhausting and time-consuming task [4]. Particularly when annotating clinical data, workers must possess not only linguistic understanding but specialized medical knowledge is also required. Recruiting such a capable workforce can make the process rather costly [5].

Furthermore, annotation is accompanied by a set of practical issues. For instance, it is natural that contributors disagree on how certain information is annotated or even whether it should be annotated [6]. Determining entity boundaries, meaning where a concept starts and ends, is one of the primary sources of conflict during the process, as so-called *boundary words*, such as articles or adjectives, can induce ambiguity [7].

**Figure 1:**
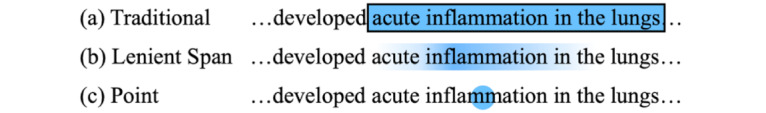
Example of different annotation paradigms. Traditional annotation (a) requires precisely labeling the beginning and end of the span, while boundary-free (b and c) methods focus on only identifying the core term.

Especially in medical texts, it is common for annotators to be unsure whether adjectives or modifiers should be included in the annotation. For example, in the sentence presented in Figure 1, some may annotate only the core symptom (“inflammation”).

Conversely, others may consider adding all modifiers necessary for a complete encapsulation of the condition.

While entity boundary definition is a problem that affects all languages, scriptio continua languages (which do not have spaces between words), such as Japanese, Chinese, and Korean, are particularly impacted due to the increased difficulty in separating concepts and modifiers.

One can employ strict annotation guidelines to delineate precisely how information should be annotated and even implement adjudication sessions to resolve disagreements. Yet, these can increase the workload and complexity of an already slow and convoluted process.

As an alternative to mitigate such issues, we propose to reformulate the annotation task by eliminating the need to define specific span boundaries when annotating an NE. By demanding less precision from the annotators, we expect to minimize the required decision-making during labeling, thus, improving annotation speed and relieving conflicts.

Although this approach may reduce annotation quality, named entity recognition (NER) performance should not be significantly impacted, as previous research found that models are resilient to a certain amount of boundary imprecision in their training data [8].

In this paper, we leverage this phenomenon by introducing 2 *boundary-free* annotation methodologies: *lenient span*, which relieves the emphasis on entity boundary precision, and *point*, which uses a single position to represent the annotation. Figure 1 presents a visual comparison between the methodologies. We performed a case study to evaluate the efficiency of the proposed methods when annotating a corpus of Japanese medical case reports to create training data for an NER system.

Our contributions are summarized as follows. We present 2 novel boundary-free annotation methodologies, evaluate the efficiency of the annotation process by metrics of annotation time and annotator agreement, and analyze the impact on the performance of an NER system trained with annotated corpora.

### Related Work

#### Annotation Efficiency Improvements

Attempts to improve the annotation workflow are a common theme in NER-related research.

Preannotation depicts the automatic labeling of the text prior to the annotator work [9]. This technique can not only reduce the required annotation time and workload required but also minimize errors [10]. Active learning (AL) [11] can further optimize automatic labeling by iteratively incorporating the data produced during the annotation process to retrain the preannotation model. Kholghi et al [12] ascertained that AL reduced the annotation time by up to 35% (5.6/16 hours) during experiments.

While these are well-established approaches, recent studies also explore alternative ideas. Tokunaga et al [13] analyzed eye-tracking data during NE annotation to identify characteristics that can help design effective features for an annotation tool. Saxena et al [14] introduced a hybrid search-enhanced software that allows users to look for similar terms and annotate related information simultaneously, shortening work time when compared to standard tools.

In recent years, generative LLMs have transformed NLP research and applications, becoming state-of-the-art NLP techniques. While the potential of LLMs to improve the text annotation workflow has also been evaluated in a few different studies [15-17], Tan et al [18] point out that their effectiveness is still strongly affected by model hallucinations and the gap in performance between proprietary and open-source LLMs.

Although crowdsourcing platforms allow the convenient annotation of vast amounts of data [19], they do not improve task execution or reduce the workload of an individual worker. In addition, as Snow et al [20] noted, inconsistent or low-quality annotations require effective quality control measures. Li [21] found that LLMs can be used to improve the quality of annotation generated by crowdsourcing. Yet LLM annotation quality is still shy of what can be produced manually; thus, combining the automated technique and human effort is still the best approach to creating a high-quality data set [22].

#### Entity Boundary Imprecision

When addressing boundary imprecision, most studies regard it as a form of noise that should be corrected or circumvented. For instance, Liu et al [23] use confidence scores and normalization techniques based on the labeling structure to estimate the correct span.

Zhu and Li [7] introduced a boundary regularization technique, redistributing a portion of the probability assigned to an annotated span to its neighboring words. This process produces a smooth transition between entity annotations and their nonentity surroundings, mitigating annotation boundary inconsistencies.

Shen et al [24] propose the NER task as a boundary-denoising diffusion process, where a model is trained to derive precise NEs from noisy spans. The authors added controlled noise to gold entity boundaries and used the imprecise data to teach a model to apply a reverse diffusion process to recover the original entity boundaries.

On the other hand, Andrade et al [8] identified that imprecise boundary annotation may not have an extensive impact in some applications. The authors evaluated the effect of various levels of imprecise boundary annotation on NER and entity linking. They identified that models are resilient to a certain amount of noise, showing a small performance drop in that range.

## Methods

### Data Set

We used the MedTxt-CR-JA corpus [25] in our experiments. This data set comprises 148 open-access case reports in Japanese. Textbox 1 presents an example document from the data set.

A case report is a detailed description of a patient’s medical condition, containing, among other information, the temporal progression of the disease and its treatment. Its format is similar to a discharge summary and is frequently used in medical NLP, such as in MIMIC-III [26] or n2c2 shared tasks [27].

This corpus was used in previous studies [28] and contains pre-existing annotations for diseases and symptom names, drugs, anatomical parts, etc. Although we discarded these labels for our experiments, we use them as a gold standard (GS) for evaluation purposes. From now on, this set of annotations is identified as the *gold standard corpus* (GSC).

**Example of a case report from MedTxt-CR-JA and its English translation.**

**Original:**

５８歳，女性．

初診の約２週間前より皮疹が出現，増悪してきたため来院した．

初診時，体幹・四肢に広範囲に浮腫性紅斑が出現し，一部では小水疱を形成していた．

手指背では関節部に一致して角化性紅斑を認め，爪囲には紅斑・紫斑を，眼周囲には軽度の紫紅色斑を認めた．

この時点ではＣＰＫ，ＬＤＨの軽度上昇，抗核抗体２０倍以外，特に異常はなく，確診に至らないため，ステロイド軟膏外用にて経過観察していたところ，３週目頃より体幹・四肢の皮疹が角化性赤色斑へと変化し，１か月目頃より上眼瞼の浮腫性紅斑が著明となり，典型疹となった．

肺癌の合併により発症１年２か月後に死亡した．

臨床経過から，初診時にみられた多形紅斑様あるいは湿疹様の皮疹を皮膚筋炎の早期皮疹と考えた

**English translation:**

A 58-year-old female.

The patient visited this hospital due to the appearance of a skin rash which worsened about 2 weeks before her first visit.

At the initial examination, the patient had extensive edematous erythema on her torso and extremities, with forming blisters.

Keratinized erythema was uniformly observed around the joints on the back side of the fingers, erythema and purpura were observed around the nails, and mild purplish-red spots were observed around the eyes.

At this point, there were no abnormalities other than mildly elevated CPK and LDH and 20-fold increase in antinuclear antibodies.

Consequently, follow-up with a topical steroid ointment was carried out.

However, by the third week, the skin rash on the torso and extremities changed to keratotic red plaques, and edematous erythema of the upper eyelids became prominent by approximately the first month and became a typical rash.

The patient died 1 year and 2 months after the onset of illness due to complications of lung cancer.

Based on the clinical history, the erythema multiforme or eczema-like skin rash seen at the time of the initial examination is considered to be an early-stage skin rash of dermatomyositis.

We randomly selected a subset of 100 documents from the full corpus, referred to from now on as the *data set*. To minimize the difference in difficulty between texts, we selected documents with similar lengths and quantity of GS entities. Texts are, on average, 554 characters long, roughly equivalent to 250-300 English words, containing around 10 entities per text.

Even though the set of documents for annotation may be considered small, it is worth noting that a scenario with such a small amount of data is not uncommon in the clinical setting, where strong data restrictions usually limit the amount of data available to work with [29].

### Annotation Guidelines

It is common to define a set of guidelines before an annotation process to minimize the divergences between annotators and guarantee consistency.

We followed the annotation schema as defined by Yada et al [30]. To simplify the evaluation process, annotators were asked to label only positive (nonnegated) entities of the “Diseases and symptoms” category. We provided the participants with a document describing what should be annotated and some examples, as summarized in Table 1.

**Table 1:** Annotation guidelines.

Description		Examples^a^
**What to annotate**		
	Reported symptoms, disease names, and clinical findings (pathology, CT^b^, and other images)	Patient visited this hospital due to the appearance of a *skin rash*.
	Clinical suspicion, even if there is a slight possibility of disease occurrence	*Epicarditis* was *suspected* and the patient was hospitalized on July 2.
	The locus of a condition, such as an anatomical structure or location, body substance, or physiologic function	Abdominal CT scan revealed *many enlarged intra-abdominal lymph nodes*.
	Adjectives and other modifier words that alter the characteristics or intensity of a condition	Patient had no subjective symptoms other than a *high fever*. There was *spotty necrosis* in the lobules.
**What should not be annotated**		
	Absence of symptoms or diseases. Basically, a negation of a clinical concept	Abdominal findings were unremarkable. The rash disappeared in about 2 months.
	General discussion of a condition merely as a reference and not as a clinical finding	There is a possibility of primary biliary cholangitis when elevated hepatobiliary enzymes are detected.
	Numeric or qualitative findings of an investigation, such as laboratory test values	The measured blood pressure was abnormal.

^a^In the examples, entities that should be annotated are marked in italics.

^b^CT: computed tomography.

### Annotation Methodologies

Our goal is to evaluate whether relieving the emphasis on entity boundary improves annotation speed while maintaining the overall quality of the produced labels. Thus, we compared the *traditional* (boundary-strict) annotation method against 2 proposed boundary-free approaches: *lenient span* and *point annotation*. Figure 1 presents a comparative example of each annotation method.

#### Traditional Annotation

Traditional annotation requires precise annotation of each NE’s exact start and end positions.

#### Lenient Span Annotation

Lenient span annotation introduces flexibility to the annotation boundaries. While the annotation is still composed of a span, start and end positions are not required to be exactly aligned with the NE boundaries.

#### Point Annotation

Unlike span-based paradigms, this method requires selecting a single point at any position within the NE span without explicitly specifying the span. It prioritizes speed and simplicity in scenarios where it is not straightforward to determine the NE span precisely. On the other hand, it may introduce ambiguity in the information captured by the annotation.

### Note on LLM Annotation

While the use of generative LLMs for text annotation is gaining traction, in this work, we seek ways to aid human annotation and reduce the necessary effort as much as possible where LLMs cannot be used.

The use of LLMs still raises concerns about privacy and security issues; as due to the necessary infrastructure and computational power needed, these models are usually held in the cloud and owned by third-party companies [31]. Given the sensitive nature of clinical data, the usage of LLMs in NLP tasks on real-world data is usually constrained by the policy of medical institutions. Thus, there is still a need for manual annotations until performant medical LLMs can be accessed through a secure private network or hosted inside hospital facilities at a reasonable cost.

### Annotation Task

We asked 4 annotators with medical background and different levels of annotation experience to participate in the experiments. They produced 3 annotated corpora by labeling the documents from the data set using each evaluated methodology. We measured the time taken for each annotation session and computed agreement metrics. We then used each produced corpus to fine-tune a Bidirectional Encoder Representations From Transformers (BERT)–based [32] NER system and evaluated its performance to assess the corpora quality.

### Annotation Tool Development

**Figure 2:**
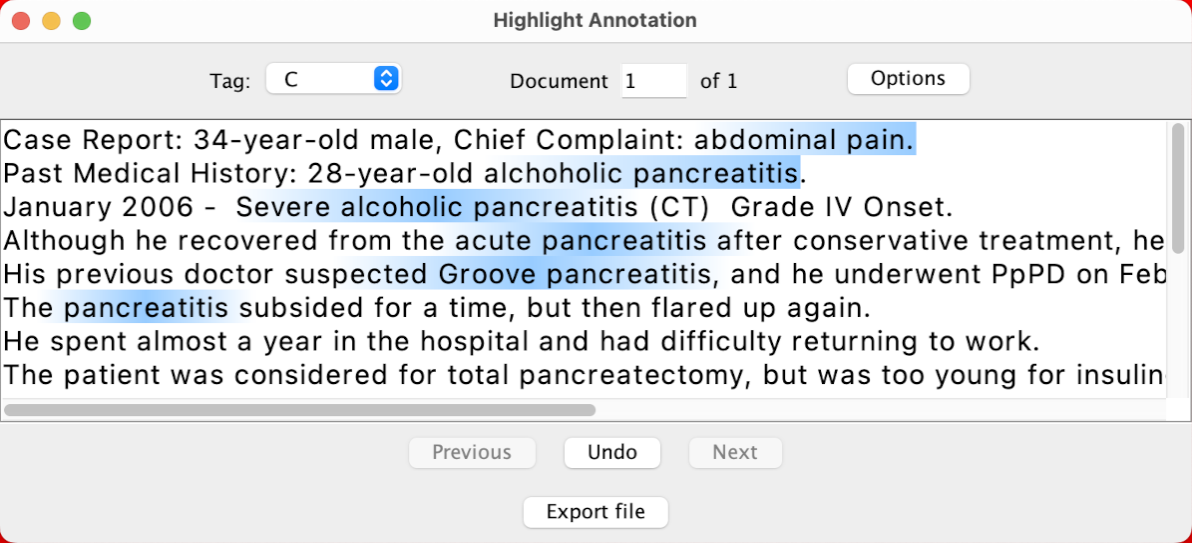
Screenshot of the annotation tool.

We developed a Java-based annotation tool to support the proposed boundary-free approaches [33]. Annotations can be presented with smoothed edges using a gradient of color to represent a *soft boundary* and encourage the annotators to be less meticulous when marking the boundaries of the concept. Figure 2 shows a screenshot of the main annotation window.

The text is displayed in its original style, keeping line breaks, spacing, and special characters. Since there is no pretokenization of the texts, annotators can select text spans with character-level precision.

The tool has the following two modes to annotate a concept: (1) *click and drag* and (2) *click-only.*

#### Click and Drag

The user clicks on the location where the concept begins and drags the mouse up to where it ends. After releasing the mouse, the area becomes highlighted, representing the labeling.

#### Click-Only

**Figure 3:**
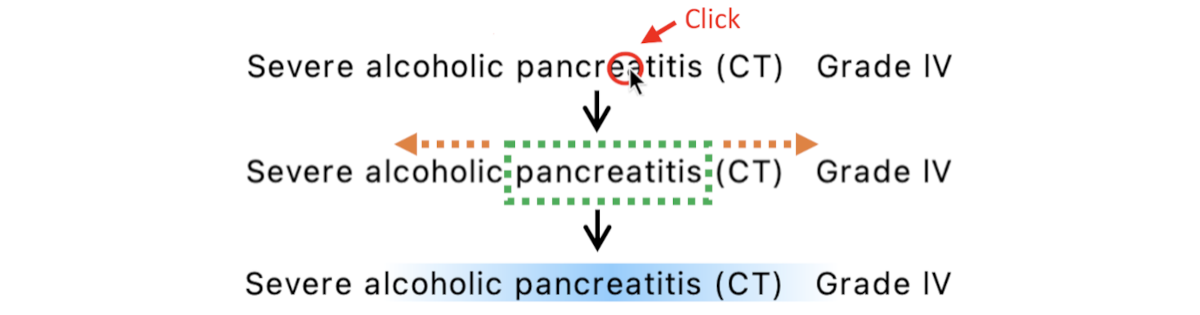
Example of a click-only annotation. The selected position, represented by the red circle, is expanded to the word boundaries (in green) plus a random span (orange arrows).

The user clicks on an entity to label it. While the annotation is stored as a single point, the position will be expanded to a *simulated* span on the interface, representing approximately the labeled concept, as shown in Figure 3.

The annotators received instructions on how to use the tool and a video demonstrating the annotation of a document. They were also supplied with 10 test documents to familiarize themselves with the tool.

### Labeling Workflow

To minimize the number of times each annotator would annotate the same document yet allow us to have at least 2 sets of annotations for a given methodology, we divided our data set of 100 documents into 4 splits.

For each annotation session, each participant received a file containing 2 splits and the annotation methodology that should be used (totaling 50 documents per annotator), as presented in Table 2.

**Table 2:** Data split for crossover experiment design.

Annotator or annotators	Documents			
	1-25	26-50	51-75	76-100
A	P^a^/T^b^	P	S^c^/T	S
B	S/T	S/T	P	P
C	S	P/T	P/T	S/T
D	P	S	S	P/T

^a^P: point annotation.

^b^T: traditional annotation.

^c^S: lenient span annotation.

We attempted to maximize the mixing between the annotator and the methodology used.

The work was executed in 3 different sessions, the first for point annotation, followed by the lenient span annotation, and lastly, the traditional annotation. During the first 2 sessions, the annotation tool was configured to show smooth edges, and annotators were instructed not to fix slightly incorrect annotations as long as the core concept was highlighted in the tool’s interface.

Although the same annotator worked on the same document more than once, the traditional annotation (third) session was conducted 6 months later to avoid memory bias affecting the annotation time measurement. This time annotators were instructed to be as precise as possible when selecting the entity spans and not to refrain from undoing incorrect annotations. The annotation tool was configured beforehand to present the annotations with precise hard boundaries, as any other standard annotation software.

Across all sessions, participants were instructed to annotate the broadest expression whenever in doubt about whether some words should be included in the annotation. Each session produced 2 parallel sets of annotations for each document, unified in a single corpus for each annotation method.

We resolved all disagreements between the 2 sets automatically. We accepted all annotations made by either annotator, even if there is no matching counterpart. Whenever there is boundary disagreement, we choose the broadest span possible when combining the 2 annotations.

For *point* annotations, we grouped annotations that refer to the same NE and averaged their positions. We consider annotations as referring to the same concept when located within 6 characters of distance from each other. The distance limit was chosen based on the average Japanese word length, around 3 characters. We chose a larger value to account for multiword concepts.

### Point-to-Span Estimation

Being aware that the single-position label produced by the *point* annotation method may not convey enough information about the adequate range of the NE to be extracted when training the model, we developed a *point-to-span* estimation method [34]. It can complement the annotation with span information without additional manual work.

**Figure 4:**
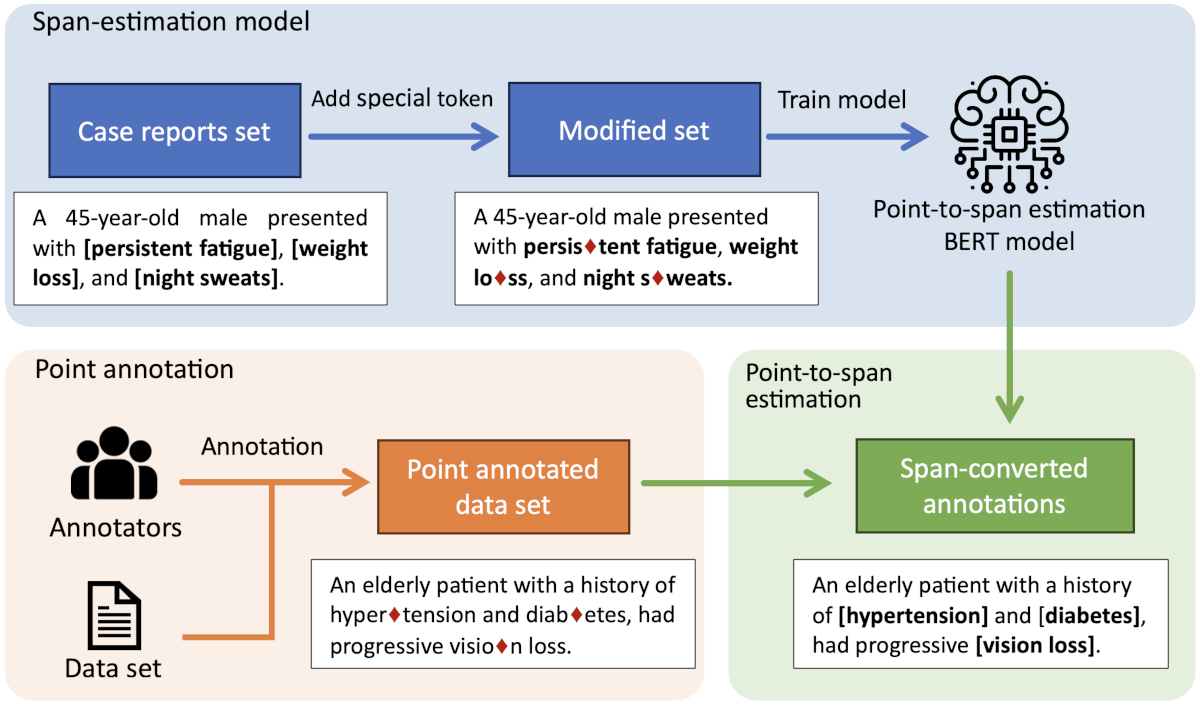
Flow of the point-to-span estimation process. BERT: Bidirectional Encoder Representations From Transformers.

We used a BERT model (referred to as the *expansion model*) that receives the positional annotation and attempts to predict the original NE span. Effectively, it works as a method to convert Points into Span-based annotations, as illustrated in Figure 4.

The *point-to-span* estimation model is based on the pretrained *tohoku-nlp/bert-base-japanese-char-v2* model [35], and it was fine-tuned using the training parameters presented in Table 3. Training was performed on a server with 2 NVIDIA Quadro RTX 8000 GPUs.

**Table 3:** Hyperparameters used for model training.

Parameter	Value
Max epochs	10
Training batch size	16
Learning rate	3×10^–5^
Optimizer	AdamW
Max sentence length	512 characters
Model selection	Early stopping
Training time	Approximately 30 min

As training data, we used a large data set of Japanese medical texts with labeled diseases and symptoms consisting of 1027 synthetic medication history notes generated through crowdsourcing. In total, 10 experienced dispensing pharmacists were hired as writers to craft the corpus. Each writer was assigned 1 of 285 drug names and tasked with creating a “typical” clinical narrative.

Before being fed to the model, each annotation of the training data was replaced by an identifier token 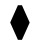 in a random location within its span based on a truncated normal distribution. A different distribution was used for each annotation, centered on the middle point, with SD being a sixth of the annotation length. Due to the randomicity of the data, we augmented the data set 10 times by re-executing the annotation replacement module and generating different valid positions for the 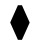.

The expansion model was then trained to identify this token and output the start and end positions of the concept based on the word containing the token and its surrounding context.

We evaluated the model by predicting the spans for annotations on the GSC. We preprocessed the GSC annotations using the same method to replace the annotations with 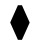. tokens. Our best model was able to achieve an *F*_1_-score of 0.77.

We applied the expansion model to the point-annotated data set to infer spans for each annotation, producing a *point-expanded* corpus. Effectively, the combination of point annotation and expansion allows the generation of a span-annotated data set with less human effort.

### Evaluation

#### Annotation Method Efficiency

We evaluated the annotation methods according to the following:

- Annotation quality: We assessed the percentage of GSC concepts that were correctly annotated. We consider an annotation correct when at least 1 token overlaps with the GS span.
- Annotation time: Annotators manually measured the time they took to work on the data during each session. They were instructed to start the timing after loading the texts in the annotation software.
- Interannotator agreement (IAA): We use Cohen Kappa [36], one of the most common metrics for gauging agreement between annotators. Kappa is a function of the proportion of observed and expected agreement, and it may be interpreted as the proportion of agreement corrected for chance [37].

Given that the *point* annotation methodology allows for multiple correct annotations within the NE span, we computed an additional *adjusted variant* of the metrics specifically for these annotations. In this variant, we considered annotations to agree if they were within a 3-character range of each other, reflecting the average word length in the Japanese language.

#### Downstream Task Performance

As one of the typical downstream tasks, we developed an NER system to benchmark each annotation approach. We again employed the pretrained *tohoku-nlp/bert-base-japanese-char-v2* model [35] and fine-tuned it using our annotated corpora.

We used the same training parameters for all models, as presented in Table 3. To minimize the variability between results, we used 5-fold cross-validation and averaged the obtained values.

We evaluated model predictions on the MedTxt-CR-JA test set, comprised of 75 documents, by the metrics of *precision*, *recall*, and *F-score*. We employ two variants of the metrics: (1) strict and (2) relaxed.

Strict metrics follow CoNLL criteria [38] and only consider predictions where the span exactly matches the ground truth. These metrics allow us to estimate how closely the model fits the GS.

Relaxed metrics [39] accept partial matches or extra tokens as long as at least 1 token of the predicted span overlaps with the GS span. This variant allows assessing the model’s capability of identifying the presence of concepts of interest in the text.

### Ethical Considerations

In this study, an annotation process was conducted with the help of human participants. All annotators were provided with detailed information about the purpose, methods, and potential uses of the data they produced, and their informed consent was obtained.

To ensure the privacy of all the patients related to the medical data used in this study, we selected a data set already fully anonymized.

As this research did not use personally identifiable information, it was exempt from institutional review board approval in accordance with the Ethical Guidelines for Medical and Health Research Involving Human Subjects stipulated by the Japanese national government (Chapter 1, Part 3, 1C) [40].

## Results

### Annotation Method Efficiency

Upon merging the data received from the annotators, we produced the final version of the annotated corpus for each one of the methodologies. Table 4 shows some statistics of the produced corpora.

There is no substantial difference between *traditional* and *lenient span* methods when comparing the average length of the produced annotation. However, both produced annotations slightly larger than the gold annotations due to the disagreement resolution approach adopted in this study.

**Table 4:** Statistics of the produced corpora.

Method	Total annotations	Average annotation length (character)
Gold standard	1167	6.31
Traditional	1065	7.30
Lenient span	1012	7.30
Point	1066	—^a^

^a^Not applicable.

#### Annotation Quality

Table 5 shows the average of GSC annotations covered by each corpus.

Although none of the methodologies captured all the ground truth concepts, the percentage of entities captured was similar for every method, with less than a 10% (73 annotations) difference between the best (lenient span) and worst (point).

As the value of missed entities is consistent for all methodologies, we attribute it to some divergence between the guidelines for annotating the GSC and the one used in this study. Differences in the interpretation may have led the annotators to skip some of the entities.

We noticed that the traditional methodology presented a more constant accuracy throughout the annotators, while the boundary-relaxed methods had more variation, especially for annotators C and D.

**Figure 5:**
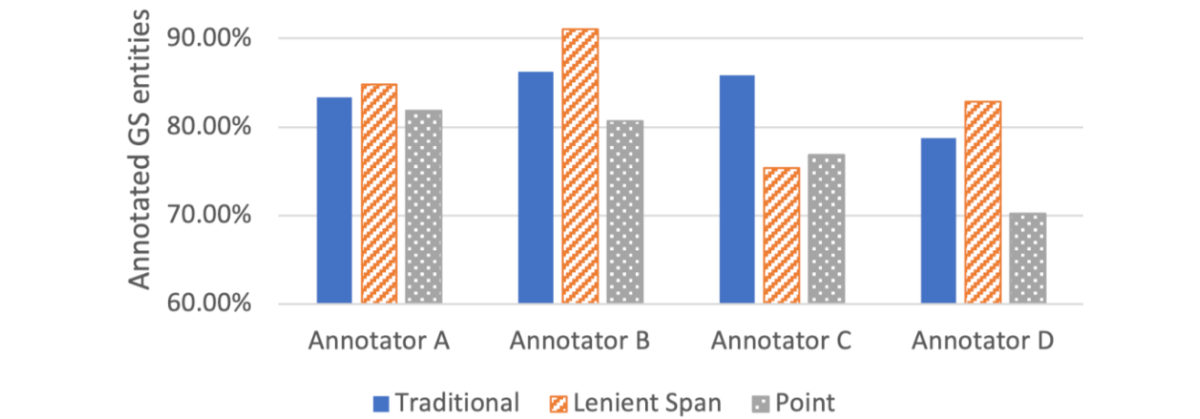
Annotation accuracy per annotator. GS: gold standard.

Figure 5 presents the accuracy of the annotations of each participant in relation to GSC on each methodology.

**Table 5:** Average number of correctly annotated gold standard (GS) entities per annotation method.

Method	Annotated GS entities, n (%)
Traditional	819 (83.56)
Lenient span	796 (83.65)
Point	746 (77.41)

#### Annotation Time

The time measurement results in Table 6 demonstrate that both boundary-free annotation techniques can provide time-saving benefits. On average, reductions of around 25% (around 28 min) and 20% (around 21 min) were observed when using *point* and *lenient span* methods, respectively, compared to the *traditional* annotation process.

**Table 6:** Comparison of the individual annotation time per annotation method^a^.

Annotator	Traditional	Lenient span	Point
A	1:23:44	1:03:23 24%)	0:54:35 (–35%)
B	1:09:14	0:52:07 (–25%)	0:48:45 (–30%)
C	3:16:58	2:10:20 (–34%)	2:15:27 (–31%)
D	1:10:23	1:31:29 (+30%)	1:10:40 (+0%)
Average	1:45:05	1:24:20 (–20%)	1:17:22 (–26%)

^a^Times are presented in the HH:MM:SS format, with the percentage comparison to the traditional method in parenthesis.

#### Interannotator Agreement

As evidenced by the results presented in Table 7, the IAA measured for both boundary-free annotation methods overcame the *Traditional* methodology.

*Point* annotations recorded the lowest agreement due to the inherent low probability of annotators precisely pinpointing the exact same position within an NE. Despite that, it achieves the highest measured agreement using the adjusted variant of the metrics.

**Table 7:** Average interannotator agreement per annotation methodology.

Method	Cohen Kappa
Traditional	0.731
Lenient span	0.774
Point	0.326
Point (adjusted)	0.811

### Downstream Task Performance

Table 8 presents the NER model evaluation results.

We trained a GSM using the GS data as a reference for our system’s best possible performance.

The data produced in our annotation experiments probably have lower quality due to the lack of proper curation and review sessions. Thus, when comparing the *Traditional* annotation approach against the GSM, there is a slight decrease in performance: 15% and 11% on strict and relaxed metrics, respectively. Nevertheless, the relation between precision and recall remains the same, as both models were trained on similarly boundary-strict annotations.

**Table 8:** Evaluation of the trained named entity recognition models.

Method	Strict			Relaxed		
	Precision	Recall	*F*_1_-score	Precision	Recall	*F*_1_-score
Gold standard model	0.72	0.78	0.75	0.90	0.89	0.89
Traditional	0.60	0.69	0.64	0.77	0.81	0.79
Lenient span	0.56	0.54	0.55	0.67	0.62	0.64
Point	0.00	0.00	0.00	0.60	0.45	0.51
Point (expanded)	0.34	0.35	0.35	0.73	0.71	0.72

## Discussion

### Principal Findings

Throughout the experiments, it was noticeable that simplifying the annotation process contributed to a more comfortable experience for the participants. We observed increased annotation speed, annotator agreement, and overall positive feedback from the annotators regarding the changes.

Although we showcase our proposal in clinical data, the annotation methodologies are both domain and language-agnostic, so they can be applied to texts of different domains and idioms.

#### Annotation Speed Improvements

The results in Table 6 show that simplifying the constraints under which annotators work can effectively increase the speed at which they execute the task. By virtually removing the need to decide on entity boundaries, both proposed methodologies allowed the annotation of our data set in less time than the *traditional* method.

However, while an overall decreasing trend in annotation time was observed, different annotators experienced varying degrees of time reduction. Notably, annotator C experienced a significant increase in efficiency when using these methodologies. Conversely, annotator D was quicker with the *traditional* annotation scheme. Still, his precision was lower than other annotators, as shown by the individual accuracy results presented in Figure 5.

#### Annotator Agreement Improvements

Meanwhile, the IAA evaluation (Table 7) revealed some interesting insights into the annotation consistency of each methodology. Both the *lenient span* and the adjusted *point* agreement overcame the *traditional* methodology by 5.88% and 10.94%, respectively.

While we believe that slightly different interpretations of what information should be annotated may have diminished *traditional* approach agreement, such a finding was still unexpected due to the higher flexibility given to the annotators when removing the need for entity boundaries. However, this improvement can be attributed to the ease with which annotators can consistently agree on the core parts of mentions (or the “main words”) compared to determining the precise boundaries of entire entities. Such boundaries may or may not encompass adjectives, modifiers, etc, which often contribute to annotation disagreements.

Notably, *point* annotations perceived a large difference in the agreement values measured using the default and *adjusted* variants of the IAA metrics. This is explained by the fact that, even though it is virtually impossible for annotators to select the same character in an NE for all annotations, they generally selected positions close to each other for the same NE. Such finding is evidenced by the distribution of annotation pairs based on the number of characters of difference between them, as depicted in Table 9.

**Figure 6:**
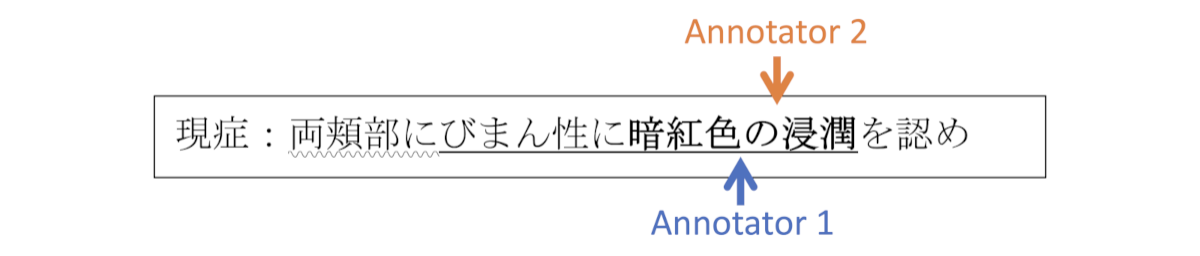
Example of 2 distinct point annotations in a named entity with a large span (underscored). The annotations are located near the center of the core word (in bold).

Such a small distance is due to annotators’ diligence in positioning the annotation close to the center of the NE’s core word. As in the sentence shown in Figure 6 (which translates to “Current symptoms: Diffuse dark red infiltration is observed on both cheeks.”), even though the span of the desired annotation is quite large, both annotators placed their labels near the most relevant set of words, “dark red infiltration.”

**Table 9:** Distribution of annotation pairs based on the distance between them.

Number of characters of difference	Annotations, n (%)
0	661 (41.31)
1	640 (40.00)
2	145 (9.06)
3	52 (3.25)
4	25 (1.56)
5	15 (0.94)
6	15 (0.94)
7	14 (0.94)
8	11 (0.69)
9	15 (0.94)
≥10	7 (0.44)

#### Annotator’s Opinions

Annotator feedback was positive especially regarding the *point* annotation, given its simplicity. The participants highlighted the easiness of the single-click selection mode, particularly due to the reduced mouse manipulation needed.

However, the participants expressed difficulty in understanding the correctness of their annotations and whether the chosen range was indeed accurate. They felt that the soft boundaries displayed by the annotation tool turned the annotations ambiguous, making them unsure whether they matched the range they intended to select.

#### Impacts on Model Performance

While achieving significant improvements in annotators’ work quality, the additional flexibility from boundary-free methods considerably impacted model performance, particularly in strict evaluation, due to the imprecise training data, as seen in Table 8.

The *lenient span*–trained model exhibited a significant subside in its recall, which hindered strict and relaxed evaluations. We did not expect that the ambiguity in NE boundaries could affect the model’s capability of locating NEs in the text.

While such performance drop may be acceptable for some applications, we believe additional annotation postprocessing methods could restore the accuracy to levels similar to the *traditional* schema.

#### Point-to-Span Estimation

In particular, the insights from *point* annotation experiments underscore the potential of automated methods to supplement human annotations. We believe that *point-to-span estimation* can be pivotal for improving annotation speed, but beyond that, it can be proven beneficial to aid in addressing other annotation problems.

Given the lackluster nature of the annotation task, it is not uncommon that annotators make mistakes, such as including punctuation markers or failing to label part of the NE simply for a lack of focus. The span estimation model can be a tool to “normalize” such annotations.

Furthermore, the estimation could be integrated into the actual annotation process by coupling it with our annotation tool, enabling the “click-only” annotation interface to present the predicted span directly and allowing the annotator to correct its mistakes.

However, there is potential for enhancements in the expansion model. Although expanding a point to the expected word seems to be a simple task, as we are evaluating our methods on a scriptio continua language, which makes the definition of the word boundaries not as obvious as in space-delimited languages, such as English.

Through analysis of the model's output, we have observed that the estimation model exhibited a tendency to choose spans larger than the GS entities, particularly when characters that act like qualitative adjectives (such as “高” for high, “急性” for acute, “巨大” for huge) were connected to the concept of interest.

For instance, the model outputted “高度の肝萎縮“ (Severe liver atrophy) instead of only “肝萎縮” (Liver atrophy). Another example was the expansion of the term “巨大な脾腎シャント“ (Giant splenorenal shunt), where 巨大な (Giant) was included.

Yet, even though the model output in these examples can be regarded as “incorrect” when compared to the GSC, from a clinical point of view, it is not uncommon that some diseases are distinguished by such modifier words. For example, “急性胆嚢炎” (acute cholecystitis) and “慢性胆嚢炎 (chronic cholecystitis), which even have different International Classification of Diseases codes, K81.0 and K81.1, respectively.

### Error Analysis

**Figure 7:**
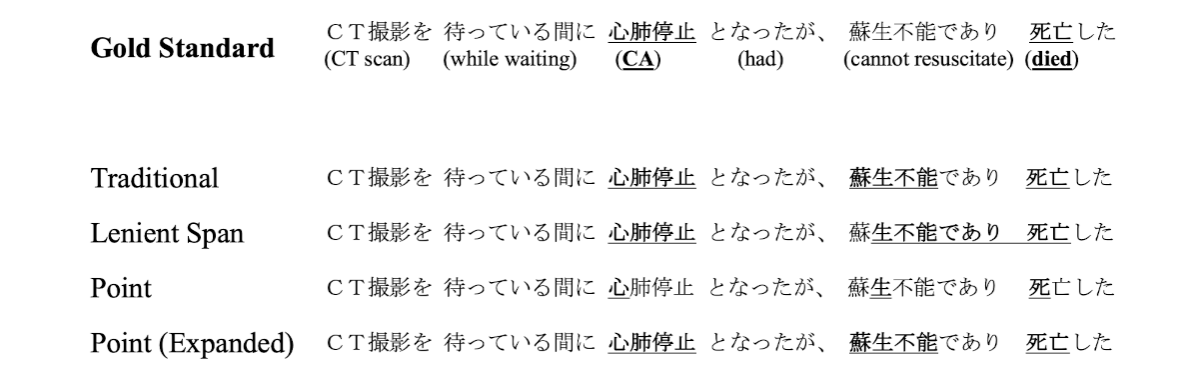
Comparison of model output for the sentence “While waiting for a CT scan, patient went into cardiopulmonary arrest (CA), but could not be resuscitated and died.” Gold standard entities and model extractions are marked in bold and underscored. White space tokenization was added to the Japanese text to enhance readability for non-Japanese readers. The original text does not contain spaces.

**Figure 8:**
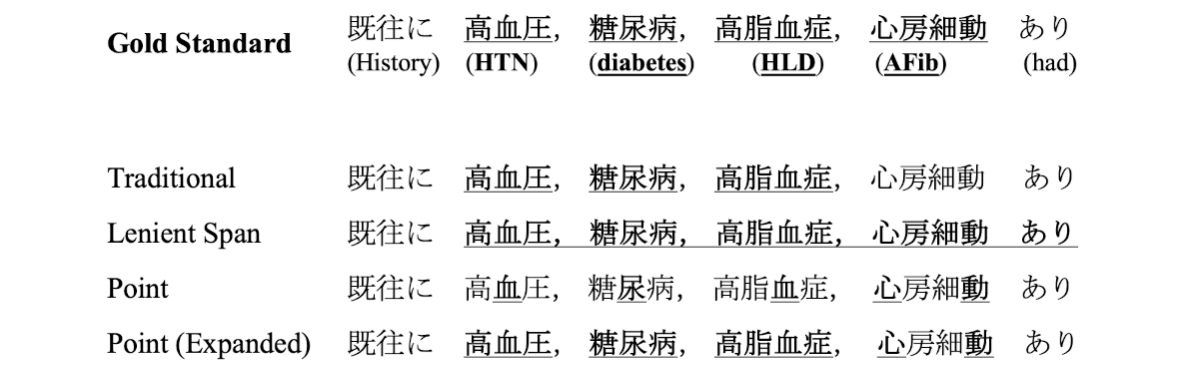
Comparison of model output for the sentence “History of hypertension (HTN), diabetes, hyperlipidemia (HLD), or atrial fibrillation (AFib).” Gold standard entities and model extractions are marked in bold and underscored. White space tokenization was added to the Japanese text to enhance readability for non-Japanese readers. The original text does not contain spaces.

Figures 7 and 8 present example comparisons between all the evaluated models in 2 different sentences.

We could not identify any unusual behavior when inspecting the traditional annotation model output. Yet, we highlight that the lenient span model portrayed a tendency to overly extend the span lengths. In some cases (as shown especially in Figure 8), multiple NEs are “merged” into a single continuous extraction.

As seen in both examples, the model trained with raw point annotations could not extract NE spans, denoting that the single position annotation contains insufficient information to train the model properly.

In contrast, the model trained on expanded point annotations showcases the effectiveness of the *point-to-span* estimation method. Although strict metrics are still substantially lower than other approaches, relaxed results are comparable to the *traditional* annotation approach. The analysis of the model output evidenced that, while it could locate most concepts of interest, it struggled in correctly extracting multiword concepts.

### Limitations

While our research focused on exploring novel approaches to text annotation and revealed promising findings, a few concerns and limitations need further investigation. Our investigations were only conducted in the Japanese language. Though our proposal is language independent, applying our techniques in a space-delimited language, such as English, could introduce some bias. Evaluation using different languages is, thus, encouraged. Since our data set in this study has an English variant, we plan to conduct additional experiments.

We concentrated on a singular entity class, disease, and symptom names to streamline the analysis. Even though our texts contain a large number of entities, a single class annotation may not represent a real use case. Exploring our methodologies in a multiclass scenario would enhance the robustness of our findings and conclusions.

Furthermore, we acknowledge that automated labeling techniques, such as preannotation, can affect the improvements observed in annotation time by adopting boundary-free methodologies. We chose not to incorporate these features in our annotation tool to minimize the number of variables affecting the annotation process.

The observed performance of the trained NER models could have been impacted by our choice of using a simple and automatic approach to solve disagreements. Although it avoids additional annotator work and simplifies the research flow, implementing adjudication or review sessions with the annotations would be preferred, as it could have provided a better annotation quality.

LLMs are prevalent in the current NLP research scenario, and their application has led to the development of systems that push state-of-the-art performance in many different tasks. In the current state of our work, we have not adopted LLMs. Still, we acknowledge that the accuracy of our methods may be improved by employing such methods in our workflow, possibly replacing the Point-to-span BERT model.

### Conclusions

In this study, we investigated the effects of reducing the emphasis on entity boundary annotations while labeling NEs in a medical data set. We proposed 2 novel boundary-free annotation methodologies, *lenient span* and *point* annotation. We evaluated the impact of their application in an annotation process regarding annotation efficiency and the quality of the labeling produced.

We also publicly released our developed annotation tool [33] and point-to-span estimation model [34].

Our results demonstrate a trade-off relation between annotation efficiency and model performance. Although not surprising, it unveils the weak points of each methodology and uncovers potential adjustments that can be made to each approach. We underscore that completely disregarding boundary information may ease the annotator’s work while it sacrifices performance to some extent.

We plan to evaluate the proposed methodologies in other languages in future work. We also intend to explore the impact of postprocessing techniques, such as normalization or boundary regularization, to enhance model output performance.

## Abbreviations

AL: active learning
BERT: Bidirectional Encoder Representations From Transformers
EHR: electronic health record
GS: gold standard
GSC: gold standard corpus
GSM: gold standard model
IAA: interannotator agreement
NE: named entity
NER: named entity recognition
NLP: natural language processing

## Data Availability

The data sets generated during and/or analyzed during this study are available in the MedTxt-CR repository [25].
